# Differential Survival for Men and Women with HIV/AIDS-Related Neurologic Diagnoses

**DOI:** 10.1371/journal.pone.0123119

**Published:** 2015-06-24

**Authors:** Martha L. Carvour, Jerald P. Harms, Charles F. Lynch, Randall R. Mayer, Jeffery L. Meier, Dawei Liu, James C. Torner

**Affiliations:** 1 Department of Epidemiology, The University of Iowa, Iowa City, Iowa, United States of America; 2 The Institute for Clinical and Translational Science, The University of Iowa, Iowa City, Iowa, United States of America; 3 The Medical Scientist Training Program, The University of Iowa, Iowa City, Iowa, United States of America; 4 Bureau of Human Immunodeficiency Virus, Sexually Transmitted Diseases, and Hepatitis, Iowa Department of Public Health, Des Moines, Iowa, United States of America; 5 Department of Internal Medicine, The University of Iowa, Iowa City, Iowa, United States of America; 6 Iowa City Veterans Affairs Health Care System, Iowa City, Iowa, United States of America; 7 Department of Psychiatry, The University of Iowa, Iowa City, Iowa, United States of America; Meharry Medical College, UNITED STATES

## Abstract

**Objectives:**

Neurologic complications of human immunodeficiency virus (HIV) infection and acquired immune deficiency syndrome (AIDS) frequently lead to disability or death in affected patients. The aim of this study was to determine whether survival patterns differ between men and women with HIV/AIDS-related neurologic disease (neuro-AIDS).

**Methods:**

Retrospective cohort data from a statewide surveillance database for HIV/AIDS were used to characterize survival following an HIV/AIDS-related neurologic diagnosis for men and women with one or more of the following conditions: cryptococcosis, toxoplasmosis, primary central nervous system lymphoma, progressive multifocal leukoencephalopathy, and HIV-associated dementia. A second, non-independent cohort was formed using university-based cases to confirm and extend the findings from the statewide data. Kaplan-Meier analysis was used to compare the survival experiences for men and women in the cohorts. Cox regression was employed to characterize survival while controlling for potential confounders in the study population.

**Results:**

Women (n=27) had significantly poorer outcomes than men (n=198) in the statewide cohort (adjusted hazard ratio=2.31, 95% CI: 1.22 to 4.35), and a similar, non-significant trend was observed among university-based cases (n=17 women, 154 men). Secondary analyses suggested that this difference persisted over the course of the AIDS epidemic and was not attributable to differential antiretroviral therapy responses among men and women.

**Conclusions:**

The survival disadvantage of women compared to men should be confirmed and the mechanisms underlying this disparity elucidated. If this relationship is confirmed, targeted clinical and public health efforts might be directed towards screening, treatment, and support for women affected by neuro-AIDS.

## Introduction

Infection with human immunodeficiency virus (HIV) and progression to acquired immune deficiency syndrome (AIDS) often result in neurologic and neuropsychiatric changes. Neurologic complications may occur in up to 40% of AIDS patients and in nearly 25% of HIV-infected persons who have not clinically progressed to AIDS [1, 2]. Certain HIV/AIDS-related neurologic conditions (neuro-AIDS), including opportunistic infections or HIV-associated malignancies affecting the central nervous system, often lead to severe disability and death and are associated with significantly higher mortality than non-neurologic AIDS [2, 3].

Despite the impact of neurologic complications on the HIV/AIDS population, the prognostic data available to affected patients and their healthcare providers have been limited, and the impact of potential factors in health care access and disparity, such as sex or gender, on survival following a neuro-AIDS diagnosis has not been well characterized. The purpose of the present study was to characterize survival for men and women from two non-independent retrospective cohorts, spanning a 27-year period of data collection in Iowa. Cohort members had one or more of the following neuro-AIDS conditions: cryptococcal meningitis, toxoplasmal meningoencephalitis, progressive multifocal leukoencephalopathy (PML), primary central nervous system lymphoma (PCNSL), and HIV-associated dementia (HAD).

## Methods

### Formation of Cohorts

The primary cohort (“statewide cohort”) was formed using surveillance data from the HIV/AIDS Reporting System (HARS and enhanced version e-HARS) maintained by the Iowa Department of Public Health. This cohort consisted of all adult patients within the database (i.e., 18 years or older at the time of the AIDS diagnosis) for whom one or more of the following conditions was recorded during the period of 1982–2008: cryptococcosis, toxoplasmosis, PML, PCNSL, and HAD (also termed “HIV encephalopathy” within the database). Follow-up data through the end of 2008 were eligible for analysis.

A university-based validation cohort (University of Iowa Hospitals and Clinics, UIHC) consisted of those individuals for whom records in both the UIHC electronic medical record and UIHC HIV Program clinical database were available during the period 1984–2009 and for whom ICD-9 codes matched one or more neuro-AIDS diagnosis: 117.5 cryptococcosis and/or 321.0 cryptococcal meningitis for cryptococcosis; 046.3 PML for PML; 200.5 primary CNS lymphoma for PCNSL (or via ascertainment from the State Health Registry of Iowa, SHRI); 130.0 toxoplasmal meningoencephalitis and/or 130.0 toxoplasmosis not otherwise specified for toxoplasmosis; and 348.39 encephalopathy, 294.11 dementia with behavior disturbance, 294.1 dementia in other diseases, 331.9 cerebral degeneration, 043.1 HIV causing CNS disease, and/or 349.9 CNS disorder not otherwise specified for HAD. Follow-up data through the end of 2009 were eligible for analysis.

Because data for the statewide and university-based cohorts could not be directly linked due to data security restrictions, the two cohorts were analyzed separately. The two cohorts were assumed to be non-independent yet non-equivalent. Specifically, the university-based cohort was expected to comprise a subsample of the cases reported to the statewide database (and, thus, a subsample of those included in the separate statewide analyses) as well as additional cases not identified by the statewide database, including some out-of-state residents.

Study procedures were approved by the University of Iowa Institutional Review Board and the Iowa Department of Public Health. Informed consent was not required for study participants, since all data were obtained retrospectively and analyzed and reported anonymously.

### Primary Endpoint and Calculation of Survival Time

For each subject, entry into the study period began on the date of diagnosis for the first reported neuro-AIDS condition for that individual. The primary endpoint for the study was all-cause mortality. Specific causes of death were not available for all cases, particularly in the statewide cohort, so analyses of neuro-AIDS-specific mortality were not attempted.

Because date of death could not be released from the statewide database due to data security restrictions, age at death was used to calculate overall survival time for this cohort, and survival time was defined as the age at death minus the age at the first neuro-AIDS diagnosis and was expressed in years. For the university-based cohort, survival time for deceased subjects was calculated using the date of death, obtained from death certificate data maintained by the State Health Registry of Iowa, and the date of the first neuro-AIDS diagnosis. Patients presumed to be alive at the end of the study period were right-censored on the final date of the study period (December 31, 2008 for the statewide cohort and December 31, 2009 for the university-based cohort), and survival or follow-up time for these individuals was calculated using the date of the first neuro-AIDS diagnosis and the final date of the study period.

### Statistical Analyses

Men and women in the statewide cohort were compared with respect to various sociodemographic and clinical factors, using Wilcoxon-Mann-Whitney and chi-square tests for continuous and categorical variables, respectively. Kaplan-Meier survival curves stratified by birth sex were prepared. The log-rank test was used to compare curves across strata, when the assumptions for this test were not clearly violated, i.e., when the curves to be compared did not intersect over the course of the follow-up period.

Cox regression was used to further characterize survival within the statewide cohort and to estimate the effect of birth sex, while controlling for potential confounders, such as reported antiretroviral therapy (ART) use, timing of neuro-AIDS (coincident with AIDS diagnosis versus later-onset), neuro-AIDS condition, age, race, and county of residence (metropolitan vs. other, defined using 2003 urban influence codes developed by the United States Department of Agriculture [4]), opportunistic infections and AIDS-related neoplasms, years since AIDS diagnosis, and HIV transmission risk group. Models with and without minimum CD4 counts, *Pneumocystis* pneumonia (PCP) diagnoses, and PCP prophylaxis data were compared. Finally, the proportional hazards assumption for each variable in the Cox regression model was verified, using an interaction term combining each variable with the natural log of survival length (time to death or end of study period, in years) [5]. Variables for which this assumption was violated (p<0.05 for the interaction term) were used as stratifying variables for a revised Cox regression model.

Similarly, for the university cohort, Cox regression modeling was used to measure the effect of birth sex, while controlling for potential confounders, such as reported ART use, neuro-AIDS condition, age, race, county of residence, comorbidities and opportunistic infections, years since neuro-AIDS diagnosis, and HIV transmission risk group. Statistical analyses were conducted using SAS version 9.2, and statistical significance was achieved whenever p<0.05.

## Results

### Characteristics of Cohorts

Survival in both cohorts was poor, with a median survival time of 1.13 years (95% CI: 0.90 to 1.86 years, 77.0% deceased, n = 225) for the statewide cohort and 3.04 years (95% CI: 1.79 to 11.62 years, 56.4% deceased, n = 172) for the university-based cohort. Both cohorts were predominantly male (88.0% in statewide cohort, 89.5% in university-based cohort), non-Hispanic white (81.8%, 86.6%), with a history of sexual contact with a male (MSM HIV transmission risk category, 66.7%, 66.3%), and residents of small metropolitan areas (67.6%, 65.5%). The median age for both cohorts was also similar (37.8 years, 36.9 years).

Sociodemographic and clinical comparisons for men (n = 198) and women (n = 27) in the statewide cohort were summarized (Table 1). The most common HIV transmission categories among women were adult heterosexual contact (n = 8, 29.6%, the predominant component of the “other” risk category for women shown in Table 1), undetermined risk (n = 8, 29.6%), and adult heterosexual contact with injection drug use (IDU) (n = 6, 22.2%), whereas among men these were MSM (n = 135, 68.2%), IDU (n = 18, 9.1%), and undetermined risk (n = 16, 8.1%). Due to a low frequency of the adult heterosexual contact risk category among men in the cohort, this category was not disaggregated from the “other” risk category for analysis. The timing of the AIDS diagnosis within the course of the epidemic differed significantly for men and women (p = 0.01), with 67.13% (n = 133) of men and 40.7% (n = 11) of women diagnosed with AIDS during the pre-HAART treatment era (i.e., prior to 1996). Women were also more likely to have a record of PCP diagnosis compared to men (n = 10, 37.0% for women and n = 40, 20.0% for men, p = 0.049).

**Table 1:** Characteristics of men and women in the statewide cohort.

		Men (n = 198)	Women (n = 27)	Men vs. Women
AIDS Age (years)		Mean = 39.1 SD = 8.8 Median = 37.9	Mean = 40.0 SD = 11.8 Median = 37.3	p = 0.87
Race				p = 0.22
	Non-Hispanic, White	166 (83.8%)	18 (66.7%)	
	Non-Hispanic, Black	18 (9.1%)	5 (18.5%)	
	Hispanic and Other	14 (7.1%)	4 (14.8%)	
Marital Status (n = 52)				p = 0.11
	Married	12 (27.9%)	5 (55.6%)	
	Other	31 (72.1%)	4 (44.4%)	
County Type*				p = 0.33
	Small Metropolitan	136 (68.7%)	16 (59.3%)	
	Other Area	62 (31.3%)	11 (40.7%)	
Transmission category				p<0.0001
	MSM	135 (68.2%)	0 (0.0%)	
	IDU	18 (9.1%)	6 (22.2%)	
	Undetermined	16 (8.1%)	8 (29.6%)	
	MSM & IDU	15 (7.6%)	0 (0.0%)	
	Other	14 (7.1%)	13 (48.1%)	
Healthcare Facility				p = 0.47
	Clinic	90 (45.5%)	9 (33.3%)	
	Hospital	61 (30.8%)	14 (51.9%)	
	Private Physician, HMO, Other	47 (23.7%)	4 (14.8%)	
OIs Other Than Neuro-AIDS**				p = 0.52
	None	128 (64.6%)	22 (81.5%)	
	One or More	70 (35.4%)	5 (18.5%)	
Neuro-AIDS Condition				p = 0.91
	Cryptococcosis	60 (30.3%)	8 (29.6%)	
	Toxoplasmosis	28 (14.1%)	5 (18.5%)	
	HAD	69 (34.9%)	10 (37.0%)	
	Mixed/PML/ PCNSL	41 (20.7%)	4 (14.8%)	
ART Use		73 (36.9%)	10 (37.0%)	p = 0.99
Pre-HAART Era^+^		133 (67.2%)	11 (40.7%)	p = 0.01
PCP Diagnosis		40 (20.0%)	10 (37.0%)	p = 0.049
PCP Prophylaxis		73 (36.9%)	10 (37.0%)	p = 0.99
Deaths		152 (76.8%)	21 (77.8%)	p = 0.91

SD = Standard deviation.

*County at AIDS diagnosis with county type derived from urban influence codes.

MSM = Male sexual contact with male, IDU = Injection drug use, “Other” includes adult heterosexual contact; HMO = Health maintenance organization; OIs = Opportunistic infections.

**Excludes *Pneumocystis* pneumonia (PCP).

^+^Diagnosis prior to 1996.

### Survival Analyses for Statewide Cohort

**Fig 1:**
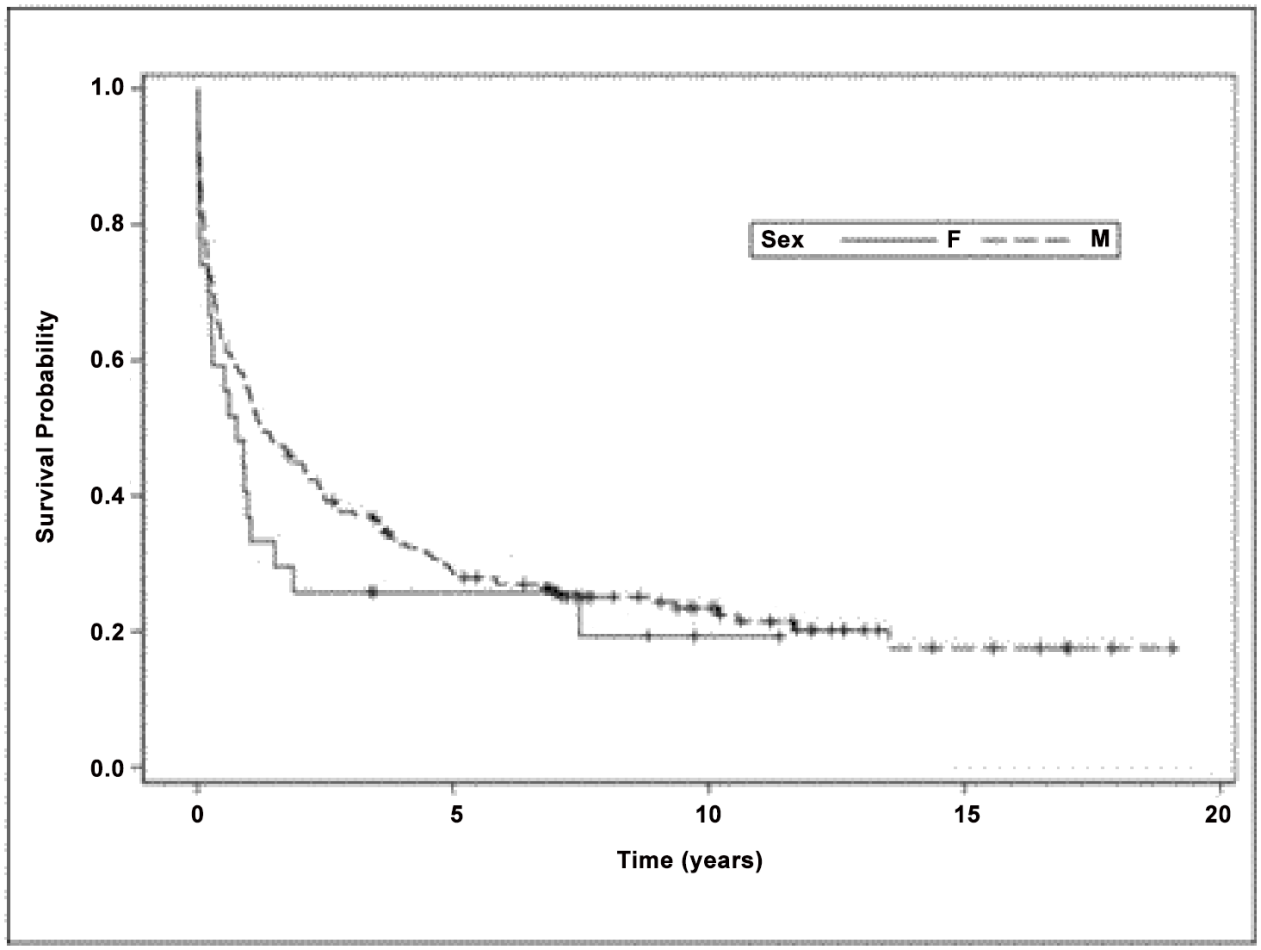
Kaplan-Meier curves for statewide cohort, stratified by birth sex.

In the statewide cohort, Kaplan-Meier analyses (Fig 1) showed the median survival time was lower for women (median = 0.78 years, range = 0.00 to 11.38 years) than for men (median = 1.30 years, range = 0.00 to 19.04 years), although this difference was not statistically significant (p = 0.13, Wilcoxon, two-tailed). In a univariate Cox regression model for the statewide model, the hazard ratio for females versus males was non-significantly elevated (HR = 1.30, 95% CI: 0.82 to 2.05), but adjustment for potential confounders unmasked a significant survival disadvantage for women compared to men (HR = 2.31, 95% CI: 1.22 to 4.35, controlling for ART by treatment era interaction, age, race, birth sex, healthcare facility type, opportunistic infection count, HIV transmission risk category, neuro-AIDS condition and stratifying on years since AIDS diagnosis and timing of neuro-AIDS, for which the proportional hazards assumption was violated).

Among the variables in the multivariate model, birth sex was most strongly confounded by the years since AIDS diagnosis variable and the HIV transmission category variable. Addition of one or both of these variables to a Cox regression model containing the birth sex variable had the greatest effect on the estimated hazard ratio for females versus males in the cohort.

### Secondary Analyses for Statewide Cohort

Overall, women in the cohort tended to have less time between the diagnoses of HIV and AIDS, lower minimum CD4 cell counts and percents, and higher maximum HIV viral loads compared to men, although only the difference in CD4 cell count was statistically significant (Table 2). Specifically, there was no significant difference in the time between the HIV diagnosis and the AIDS diagnosis (p = 0.51, total n = 213), with a median value of 0.58 years for females and 0.67 years for males. Also, while completeness of CD4 laboratory values was similar for females (minimum CD4 cell count, n = 17/27, 63.0%) and males (minimum CD4 cell count, n = 127/203, 62.6%), the median minimum CD4 cell count differed significantly between these two groups, with a value of 38.0 cells/μL for males and 19.0 cells/μL for females (p = 0.03). The minimum CD4 cell percent was not significantly different between groups (p = 0.27), although males trended towards a higher percentage (median of 5% vs. 3% for females). Similarly, completeness of viral load values was comparable for both groups (n = 10, 37.0% for females vs. n = 64, 31.5% for males), and the maximum HIV viral load was considerably higher for women than for men (median maximum of 141,548 copies/mL and 53,180 copies/mL for women and men, respectively), although this achieved only borderline significance (p = 0.08).

**Table 2:** Comparison of measures of disease severity by birth sex.

	Women (n)	Men (n)	Women vs. Men
Median Time Between HIV and AIDS (years)	0.58 (26)	0.67 (187)	p = 0.51
Median of Minimum CD4 Count (cells/μL)	19.0 (17)	38.0 (127)	p = 0.03
Median of Minimum CD4 Percent	3.0 (17)	5.0 (112)	p = 0.27
Median of Maximum HIV Viral Load (copies/mL)	141,548 (10)	53,180 (64)	p = 0.08

In a multivariate Cox regression model controlling for PCP diagnosis and prophylaxis as well as the covariates listed previously, the hazard ratio for women compared to men was 2.22 (95% CI: 1.14 to 4.33, n = 225), whereas this was 2.44 (95% CI: 0.96 to 6.22, n = 139) in the model containing minimum CD4 cell count. In secondary analyses, there was no evidence of a differential effect of ART by birth sex (interaction term p = 0.97) and no evidence that the effect of birth sex changed over time (interaction term p = 0.36). Similarly, no interaction was identified between birth sex, ART, and treatment era (p = 0.39). However, the effect of birth sex was modified by the county of residence at the time of the AIDS diagnosis. Women in small metropolitan areas had significantly worse outcomes than men in small metropolitan areas, while differential survival among men and women in less populous areas was not as pronounced (Table 3).

**Table 3:** Interaction of birth sex and county type (n = 225).

Birth Sex by County Status		HR (95% CI)*
Men	Metropolitan Area (n = 136)	reference
	Other Area (n = 62)	1.20 (0.80, 1.79)
Women	Metropolitan Area (n = 16)	3.85 (1.77, 8.37)**
	Other Area (n = 11)	1.34 (0.51, 3.52)

*Hazard ratio (HR) with 95% confidence interval (CI), adjusted for ART by treatment era interaction, age, race, birth sex, healthcare facility type, opportunistic infection count, HIV transmission risk category, and neuro-AIDS condition and stratifying on years since AIDS diagnosis and timing of neuro-AIDS.

**p = 0.0007.

Note: County at AIDS diagnosis was derived from urban influence codes. “Other area” denotes any county not designated by urban influence codes as a small metropolitan area.

Exclusion of men with the longest survival times in the cohort did not appreciably affect the effect of birth sex on survival. A total of 14 males (6.9% of the male stratum) with the longest survival times in the cohort had to be excluded in order for the elevated risk of females compared to males to lose statistical significance (HR = 1.83, 95% CI: 0.97 to 3.46, n = 211, model restricted to survival times <12 years).

### Analyses for University-Based Cohort

In the university-based cohort, the rate of death was non-significantly higher for women than for men (adjusted HR = 1.44, 95% CI: 0.44 to 4.72, n = 171). There was no evidence of interaction between sex and county type (p = 0.74), ART use (p = 0.58), or years since the neuro-AIDS diagnosis (p = 0.16).

A diagnosis of depression (including depressive disorders, psychotic depression, and bipolar depression) was documented in the university-based medical record for 27 patients (5.6% of women in the cohort and 17.0% of men), although 26 of these patients were male. The effect of birth sex on survival remained elevated (adjusted HR = 1.31, 95% CI: 0.40 to 4.34) in a model accounting for documented depression. Similarly, inclusion of marital status (married vs. other), a variable available only from the death record for this cohort, did not appreciably alter the results (adjusted HR, 1.97, 95% CI: 0.42 to 9.28, n = 94).

## Discussion

Neurologic and neuropsychiatric complications of HIV infection affect a substantial proportion of HIV-infected patients, and severe complications frequently result in significant disability or death. The advent of HAART, combined with other medical advances and psychosocial support mechanisms available to affected patients, has markedly improved survival for HIV-infected patients in recent years. However, HIV-associated neurologic manifestations remain a subject of active research interest in the HAART era, as a growing body of literature now demonstrates that HIV infection can exert diverse influences on the central and peripheral nervous systems and can produce both acute and chronic changes in affected patients [6, 7].

In this study, women in both a statewide, population-based cohort and a non-independent (yet non-equivalent), university-based cohort with neuro-AIDS tended to fare worse than their male counterparts, and this disadvantage was statistically significant in the statewide cohort. This finding was similar for both cohorts, although the results may be limited by the low numbers of women in the study (n = 27 in the statewide sample and n = 17 in the university-based sample). Even so, the consistent detection of this disparity in both cohorts, representing more than 25 years of data collection, warrants further investigation of the survival experience of male and female patients with neuro-AIDS.

Several potential factors in the observed effect of birth sex were considered here, including potential differences in initial disease severity, timing within the overall HIV/AIDS epidemic, effects of ART, adherence to ART, neurologic or psychiatric comorbidity, and psychosocial support.

On average, women in the statewide cohort presented with more severe disease than men. However, in both cohorts, inclusion of measures of severity such as opportunistic infection or comorbidity counts, PCP data, and CD4 cell values did not eliminate the apparent survival disadvantage for women compared to men, and in many such instances, the effect of birth sex remained statistically significant. Although it is likely that the initial severity of AIDS and/or neuro-AIDS among women at the time of diagnosis contributed to the observed disparity, it does not appear to explain the full effect of birth sex on survival following a neuro-AIDS diagnosis. Furthermore, a substantial difference between men and women with respect to disease severity at the time of the initial neuro-AIDS diagnosis would itself constitute a notable disparity and one which would likely result in poorer outcomes among affected women.

Women represent a second wave of the HIV/AIDS epidemic, offset from the recognized start of the epidemic in men and, therefore, occurring in parallel to advances in therapeutics and other resources for HIV/AIDS. Although women still represent a minority of HIV/AIDS patients, the proportion of AIDS cases affecting U.S. women had risen from less than 10% in 1985 to around 25% prior to the advent of HAART therapy in 1996 [8]. As a result, women in both cohorts may have been more likely to be diagnosed at a time when survival rates among HIV/AIDS patients were also climbing. In this study, controlling for year of diagnosis (as well as HIV transmission risk category) unmasked a significant survival difference between men and women in the statewide cohort, a finding which underscores the importance of multivariable modeling (or other methods to control for year of diagnosis) in studies of male and female patients with HIV/AIDS.

Previous investigations of HIV/AIDS have presented conflicting evidence about the effect of birth sex on survival and disease progression overall. Studies have reported no difference in survival between men and women with HIV/AIDS [9–15], no difference in disease progression but a significant survival disadvantage for women compared to men [16], and no difference in survival among men and women taking ART but worse outcomes for females compared to males in a non-ART group [17]. Another study demonstrated marginal protection against disease progression and death among women despite higher average CD4 counts at the time of seroconversion for HIV, progression to AIDS, and death for female patients [18]. Elsewhere, researchers have described protection against progression to AIDS and death for women compared to men [19], particularly during the HAART treatment era [20], while a 2001 study suggested that HAART-era survival benefits were actually attenuated for women compared to men [21]. A review of gender differences in HIV/AIDS found no conclusive evidence of survival disparities among men and women with HIV [22].

The present study explored the effect of sex on survival among a specific subset of HIV/AIDS patients—that is, among those with neurological complications of the disease. The results of this study demonstrate that, controlling for year of diagnosis, HIV transmission risk category, ART, and other potential confounders, women had poorer outcomes with respect to all-cause mortality than men with neuro-AIDS. Furthermore, there was no evidence that this improved over time (no interaction between birth sex and year of diagnosis), as the risk of HIV/AIDS among women became more widely recognized.

In addition, the present study provides no evidence that the prognostic effect of ART itself differed between men and women. These results may suggest that ART exerts similar neurologic or immunologic effects among men and women, at least as such effects pertain to overall survival following a neuro-AIDS diagnosis, and/or may reduce concern that ART adherence appreciably differed among men and women. Interestingly, Kempf et al. showed that men and women in a university-based retrospective cohort study of HIV/AIDS patients were equally likely to change or discontinue ART but that women, on average, spent more days off medication compared to men, and that women were also more likely to change or discontinue medications due to poor adherence and/or drug toxicities, including neurologic and psychiatric symptoms [23].

This latter finding by Kempf et al. may suggest that neuropsychiatric symptoms or comorbidities occur at higher rates among women, which may in turn have detrimental impacts on survival. Research elsewhere suggests that progression of nervous system disease in HIV/AIDS, including performance on neuropsychological assessments as well as plasma and cerebrospinal fluid viral RNA, does not differ significantly between HIV-infected men and HIV-infected women [24]. Meanwhile, investigators have also begun to explore the role of estrogen in neuroprotection and immune modulation in neuro-AIDS [25].

The presence of specific comorbidities, such as major depression, particularly if such diagnoses are not correctly identified among affected female patients, may help to explain a survival disparity between men and women. Here, controlling for documented depression in the university-based cohort did not reverse the effect of birth sex on survival in a Cox regression model, albeit with a very small number of cases (including only one woman with documented depression). Notably, the prevalence of documented depression in this study (5.6% of women in the cohort and 17.0% of men) differs from that reported in other cohorts (56.1% of HIV-positive women with depressive symptoms in the Women’s Interagency HIV Study compared to 21.0% of HIV-positive men in the Multicenter AIDS Cohort Study [26]), suggesting that depression may have been underascertained among female neuro-AIDS patients in this study and/or that neuro-AIDS severity and progression in female patients precluded psychiatric diagnoses. Notably, a recent cohort study assessing the impact of ART on clinically significant depression in the Spanish AIDS Research Network also found no statistically significant difference in the incidence of depression between male and female participants, although there was a non-significant trend favoring a higher incidence among women (adjusted incidence rate ratio = 1.45, 95% CI: 0.96–2.18, p = 0.074) [27]. While the findings regarding sex, depression, and survival in the current study should be interpreted with caution, additional research should certainly be undertaken to explore this relationship further.

Finally, the survival disadvantage for women in the statewide cohort predominantly occurred among men and women who lived in small metropolitan areas. This, combined with a non-significant impact of county status itself in either cohort, suggests that geographic access to care did not contribute to the survival disadvantage for women. Furthermore, men and women in rural environments and women in small metropolitan areas all tended to fare worse than men in small metropolitan areas (although only the difference between men and women in metropolitan areas was statistically significant). This may reflect improved systems of psychosocial support for those who currently bear the predominant share of the HIV/AIDS disease burden in the state, with less support for those who fall outside of the average patient profile. It is also possible that the interaction of birth sex and county of AIDS diagnosis represents a migration effect—that women with more substantial comorbidities, including psychiatric comorbidities, or fewer psychosocial or socioeconomic resources (perhaps even as a result of medical illness), were more likely to reside in or present for care in a small metropolitan area.

Lastly, although inclusion of the marital status variable in the university-based model may further represent the role of psychosocial support in survival among women and men, this result should also be interpreted with caution for multiple reasons. First, it involved only a small number of cases. Second, marital status was ascertained from the death document alone and does not represent persons who survived until the end of the study period. Finally, this variable likely provides an inaccurate representation of relationship support among many persons in the MSM category, for whom marriage to male partners was less probable due to legal restrictions throughout early portions of the study period.

## Conclusion

This study demonstrates an apparent survival disadvantage for women compared to men with neuro-AIDS. Initial disease severity and/or psychosocial factors may partially explain this effect. The observed survival disadvantage of women compared to men with neuro-AIDS should be confirmed in other patient populations. The mechanisms underlying any such disparity, including the clinical progression of disease and the role of neuro-AIDS-specific causes of death, should also be further elucidated. To accomplish this, efforts to include women, as well as members of other underrepresented groups with respect to the HIV/AIDS population as a whole, should be widely encouraged in neuro-AIDS research.
